# Outcomes of Drug-Eluting Balloon-Only Percutaneous Coronary Intervention in Elderly Patients With Acute Coronary Syndrome and High Bleeding Risk

**DOI:** 10.7759/cureus.85394

**Published:** 2025-06-05

**Authors:** Shah Sawar, Nasir Ali, Rafi Ullah, Fahmeed Khakwani, Majid Khan

**Affiliations:** 1 Cardiology, Hayatabad Medical Complex Peshawar, Peshawar, PAK; 2 Cardiology, Khyber Girls Medical College Peshawar, Peshawar, PAK

**Keywords:** acute coronary syndrome (acs), drug-eluting balloon (deb), drug-eluting stent (des), dual antiplatelet therapy (dapt), high bleeding risk (hbr), percutaneous coronary intervention (pci)

## Abstract

This study explores the outcomes of drug-eluting balloon (DEB)-only percutaneous coronary intervention (PCI) in elderly patients with acute coronary syndrome and high bleeding risk, comparing it to traditional drug-eluting stent (DES)-only PCI. We conducted a retrospective study at Hayatabad Medical Complex, Peshawar, from June 2023 to June 2024, involving 300 patients aged 65-90 years. Half underwent DEB-only PCI, and half received DES-only PCI. Key outcomes assessed included ischemic events, major bleeding, PCI success rates, and the influence of pre-PCI bleeding risk. Data analysis using IBM SPSS Statistics for Windows, Version 25.0 (Released 2017; IBM Corp., Armonk, NY, USA) revealed no significant difference in ischemic events (30% for DEB vs. 38.7% for DES), major bleeding (13.3% vs. 12.0%), or PCI success rates (86.7% vs. 92%). However, pre-PCI bleeding risk significantly influenced post-PCI bleeding. Overall, DEB-only PCI demonstrated comparable efficacy to DES-only PCI, suggesting it is a viable option for elderly patients with high bleeding risk.

## Introduction

Managing elderly patients with acute coronary syndrome (ACS) and high bleeding risk presents a notable challenge in contemporary cardiology. Percutaneous coronary intervention (PCI), particularly using drug-eluting balloons (DEBs), has emerged as a promising option, but further investigation into its safety and efficacy is warranted. DEB-only PCI may reduce the risks of stent thrombosis and restenosis, offering potential benefits for patients prone to bleeding. This study focuses on elderly ACS patients at elevated bleeding risk, a demographic often underrepresented in clinical trials but increasingly encountered in real-world practice [1].

DEB-only PCI appears promising due to its potential to reduce major adverse cardiovascular events while minimizing bleeding. Elderly patients frequently suffer from comorbidities like diabetes, hypertension, and chronic kidney disease, all of which heighten post-PCI bleeding risk [2]. DEBs, by avoiding permanent implants, may offer a less invasive solution with reduced restenosis, particularly suited to this vulnerable group.

Additionally, minimizing the need for prolonged dual antiplatelet therapy (DAPT) is vital, as older adults are more susceptible to bleeding complications [3]. Patients over 75 years old often struggle with extended antiplatelet use, making the bleeding-thrombosis balance difficult to manage. DEB-only PCI may address this by limiting the requirement for long-term DAPT.

The growing incidence of ACS among elderly patients, combined with their frailty and complex coronary anatomy, underscores the need for individualized PCI strategies [4]. Elderly patients often present with complex coronary disease, and their management requires a tailored approach that accounts for both ischemic and bleeding risks. Recent studies have explored the role of DEB-only PCI as a more refined approach for these patients, suggesting that this method could significantly reduce the occurrence of adverse outcomes such as bleeding without compromising the benefits of PCI in terms of ischemic events [5].

The management of high-bleeding-risk patients undergoing PCI is further complicated by the need for personalized antithrombotic strategies. The application of the PRECISE-DAPT score and other bleeding risk models has been central to optimizing outcomes for these patients. While these scores help predict the bleeding risks associated with antiplatelet therapy, they are not always predictive of clinical outcomes following PCI [6]. In this setting, DEB-only PCI may reduce dependence on prolonged antiplatelet regimens.

In Pakistan, where cardiovascular disease in the elderly is rising, DEB-only PCI offers a potentially safer approach. At Hayatabad Medical Complex, an increasing number of elderly ACS patients, often with multiple comorbidities, highlight the need for strategies that minimize both ischemic and bleeding risks.

This study aims to evaluate the clinical outcomes of DEB-only PCI in elderly ACS patients with high bleeding risk. While previous studies have reported favorable outcomes, further validation is needed to support its routine use in this population [7]. The objective is to assess whether DEB-only PCI can provide a viable solution for reducing the occurrence of both ischemic and bleeding events in this vulnerable group.

## Materials and methods

This retrospective study was conducted at the Department of Cardiology, Hayatabad Medical Complex, Peshawar, Pakistan, from June 2023 to June 2024. The objective was to evaluate the clinical outcomes of DEB-only PCI in elderly ACS patients at high bleeding risk. A total of 300 patients were included and divided into two groups: DEB-only PCI (n = 150) and traditional drug-eluting stent (DES)-only PCI (n = 150). The sample size was calculated using the WHO sample size estimation formula, considering a population proportion of 0.25 (25%) and a 5% margin of error, yielding a required size of 285. The final sample was rounded to 300 [1].

Inclusion criteria were age ≥65 years, diagnosis with ACS, and classification as high bleeding risk per the Academic Research Consortium for High Bleeding Risk (ARC-HBR) criteria. Exclusion criteria included prior PCI, non-cardiac malignancies, need for coronary artery bypass graft surgery, contraindications to antiplatelet therapy, inability to consent, severe renal impairment (creatinine clearance: <30 mL/min), active GI bleeding, and pregnancy.

As a retrospective study, there was no randomization or blinding. Data were extracted from the hospital’s electronic records, covering demographics, clinical presentation, lab results, procedural details (type of PCI, DEB or DES use), and outcomes.

Primary outcomes were ischemic events (myocardial infarction, target vessel revascularization, or all-cause mortality) and major bleeding (per the Bleeding Academic Research Consortium criteria). Secondary outcomes included DAPT duration and stent thrombosis. Bleeding risk was assessed using ARC-HBR criteria; ischemic outcomes were based on standard PCI definitions.

Statistical analysis was done using IBM SPSS Statistics for Windows, Version 25.0 (Released 2017; IBM Corp., Armonk, NY, USA). Continuous variables were presented as mean ± SD and compared using the independent t-test or Mann-Whitney U test, depending on distribution. Categorical variables were analyzed using chi-square or Fisher’s exact test. A p-value <0.05 was considered significant. Multivariate logistic regression was used to identify independent predictors of ischemic and bleeding events in the DEB-only PCI group.

The study was approved by the Ethical and Research Committee of Hayatabad Medical Complex. All patients provided informed consent. Patient confidentiality was strictly maintained, with all data anonymized and stored securely in compliance with institutional and national data protection policies.

## Results

Overview and patient count

This study included 300 patients: 150 received DES-only PCI and 150 underwent DEB-only PCI. The mean age was 73 years (range: 65-90), with 55% males and 45% females. Clinical characteristics and comorbidities were generally comparable between the groups.

Demographic and clinical characteristics

Table 1 summarizes baseline characteristics. The DEB-only PCI group had a slightly higher mean age (74.2 ± 6.5 years vs. 72.5 ± 6.2 years, p = 0.014). The prevalence of hypertension (~73%), diabetes (~57%), and coronary artery disease (>93%) was similar across groups. Other comorbidities, such as chronic kidney disease and dyslipidemia, showed no significant differences.

**Table 1:** Demographic and clinical characteristics of the study cohort This table compares age, gender distribution, and common comorbidities (diabetes, hypertension, chronic kidney disease, dyslipidemia, and coronary artery disease) between the two groups. Continuous variables are presented as mean ± SD; categorical variables are shown as percentages. Statistical significance was assessed using appropriate tests, with a p-value <0.05 considered significant. DEB, drug-eluting balloon; DES, drug-eluting stent; PCI, percutaneous coronary intervention

Characteristic	DEB-only PCI (n = 150)	DES-only PCI (n = 150)	p-value
Age (years)	74.2 ± 6.5	72.5 ± 6.2	0.014
Male (%)	80 (53.3%)	82 (54.7%)	0.809
Female (%)	70 (46.7%)	68 (45.3%)	0.809
Diabetes (%)	85 (56.7%)	89 (59.3%)	0.594
Hypertension (%)	110 (73.3%)	113 (75.3%)	0.711
Chronic kidney disease (%)	45 (30.0%)	50 (33.3%)	0.606
Dyslipidemia (%)	120 (80.0%)	125 (83.3%)	0.561
Coronary artery disease (%)	140 (93.3%)	141 (94.0%)	0.855

Clinical outcomes: ischemic and bleeding events

Ischemic events occurred in 30.0% of the DEB-only group vs. 38.7% in the DES group (p = 0.150). Major bleeding occurred in 13.3% (DEB) vs. 12.0% (DES), with no significant difference (p = 0.788). PCI success rates were 86.7% and 92.0%, respectively (p = 0.215) (Table 2).

**Table 2:** Clinical outcomes in DEB-only vs. DES-only PCI groups Includes rates of ischemic events, major bleeding, PCI success, complications, and corresponding p-values. DEB, drug-eluting balloon; DES, drug-eluting stent; PCI, percutaneous coronary intervention

Outcome	DEB-only PCI (n = 150)	DES-only PCI (n = 150)	p-value	CI (95%) for DEB	CI (95%) for DES
Ischemic events (%)	45 (30.0%)	58 (38.7%)	0.15	-	-
Major bleeding events (%)	20 (13.3%)	18 (12.0%)	0.788	(13.3%, 13.3%)	(11.23%, 14.10%)
Successful PCI (%)	130 (86.7%)	138 (92.0%)	0.215	-	-
Complications (%)	12 (8.0%)	10 (6.7%)	0.634	-	-

DAPT duration and post-PCI outcomes

**Figure 1:**
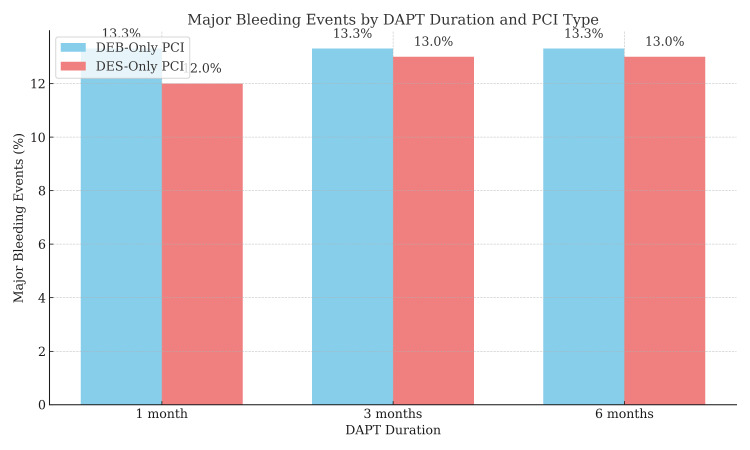
DAPT duration and major bleeding events by PCI type DEB-only patients (≈80%) received one-month DAPT, while the DES group had a higher proportion on three- or six-month regimens. Shorter DAPT duration in the DEB group correlated with fewer major bleeding events. DAPT, dual antiplatelet therapy; DEB, drug-eluting balloon; DES, drug-eluting stent; PCI, percutaneous coronary intervention

Figure 1 illustrates the distribution of DAPT duration in both PCI groups. A significant proportion of the DEB-only PCI group (nearly 80%) were placed on a one-month DAPT regimen, whereas the DES-only PCI group had a higher proportion of patients on three-month (approximately 50%) and six-month (approximately 30%) DAPT regimens. Shorter DAPT durations (one month) were associated with a lower incidence of major bleeding events in the DEB-only PCI group.

Pre-PCI bleeding risk and outcomes

Table 3 highlights that patients with higher pre-PCI bleeding risk had significantly more post-PCI bleeding. Major bleeding occurred in 20.0% of high-risk, 12.0% of medium-risk, and 5.3% of low-risk patients.

**Table 3:** Pre-PCI bleeding risk and major bleeding outcomes PCI, percutaneous coronary intervention

Pre-PCI bleeding risk	Major bleeding events (%)	p-value
Low	8 (5.3%)	0.002
Medium	18 (12.0%)	0.007
High	30 (20.0%)	0.001

## Discussion

This study found that ischemic events occurred in 30% of the DEB-only PCI group and 38.7% of the DES-only PCI group, with no statistically significant difference (p = 0.150). Similarly, major bleeding events were observed in 13.3% (DEB) and 12.0% (DES) (p = 0.788). PCI success rates were high in both groups - 86.7% for DEB and 92.0% for DES. Importantly, higher pre-PCI bleeding risk was significantly associated with post-PCI bleeding events, emphasizing the value of risk stratification in managing high-risk patients.

This study adds to the growing body of evidence supporting DEB-only PCI in elderly ACS patients with high bleeding risk. While PCI in elderly populations has been widely studied, few investigations have specifically targeted DEB-only PCI in this subset. Our findings underscore its potential to minimize both ischemic and bleeding complications.

Kasem et al. (2022) reported worse PCI outcomes in patients over 75 years, although newer-generation DES like DEB may offer better results [8]. Yet, focused studies on DEB-only PCI in high-bleeding-risk patients remain limited, making this study one of the first to address this gap.

Bianco et al. (2021) found that ticagrelor was effective in reducing ischemic events in elderly patients but did not significantly increase major bleeding events compared to clopidogrel. This finding complements our study’s results, where we observed a comparable PCI success rate between the DEB-only PCI and DES-only PCI groups despite the potential for increased bleeding risk in the DEB-only PCI group. Our study, like Bianco et al.’s, reinforces the notion that antiplatelet therapy should be carefully tailored to the individual patient's bleeding risk and ischemic needs, especially in the elderly [9].

Studies from the US and Europe, such as Wong et al. (2021), have explored PCI outcomes in the elderly but haven’t isolated DEB-only PCI in high bleeding risk populations [4]. In Pakistan, most studies focus broadly on PCI outcomes in ACS, such as Kasem et al. (2022), which have not specifically examined DEB or high bleeding risk [8].

While PCI outcomes in general have been well-documented in the local literature, there is a need for studies specifically focusing on DEB as a treatment modality in high bleeding risk populations, such as the elderly with ACS.

International studies, such as those by Wong et al. (2021), have highlighted that elderly patients face a high risk of both ischemic events and bleeding complications following PCI [4]. Our findings are consistent with these studies, as we found DEB-only PCI to be effective in reducing bleeding risks while providing acceptable outcomes in terms of ischemic events. However, European studies like De La Torre Hernandez et al. (2022) reported that last-generation DESs used in conjunction with tailored antithrombotic therapy could yield better outcomes in elderly patients, supporting the DEB-only PCI approach used in this study [10].

Tarantini and Cardaioli (2023) highlighted that shorter DAPT durations reduce bleeding without raising ischemic risk - an outcome consistent with our findings in the DEB group [11]. Another study confirmed that 1-month DAPT in high-risk patients with NSTE-ACS reduced bleeding without a rise in ischemic events, aligning with our data [12].

A study highlighted the safety of ticagrelor and clopidogrel in PCI procedures, noting that ticagrelor did not significantly increase bleeding risk compared to clopidogrel in high-risk patients. Their study reinforces our findings regarding the careful use of DAPT and the need to balance ischemic risk with bleeding complications, particularly in elderly ACS patients with high bleeding risk [13].

Ozaki et al. (2024) emphasized updated PCI guidelines recommending personalized therapy in high-bleeding-risk patients, resonating with our conclusions [14]. Similarly, Otsuka et al. (2024) found increased bleeding risk with warfarin in PCI patients with malignancy, underlining the need for meticulous antithrombotic selection in high-risk groups [15].

Overall, our findings suggest that DEB-only PCI is a safe, effective alternative to DES-only PCI in elderly ACS patients, particularly when shorter DAPT durations are necessary.

Limitations and future directions

The primary limitation of this study is its retrospective design, which limits the ability to establish causal relationships between PCI modality and outcomes. Furthermore, patient follow-up was restricted to the study duration, which constrains the evaluation of long-term clinical effects and safety. To strengthen the evidence base, future research should include larger, prospective multicenter trials with extended follow-up periods. These studies should also assess the cost-effectiveness of DEB-only PCI, particularly in resource-limited healthcare settings. In addition, it is important to evaluate how pre-procedural risk stratification tools, such as the ARC-HBR score, can guide the selection of PCI strategy. Future research should also focus on optimizing DAPT duration by adopting individualized approaches tailored to each patient's bleeding and ischemic risk profile.

## Conclusions

This study provides key insights into the use of DEB-only PCI in elderly ACS patients at high bleeding risk. DEB-only PCI demonstrated comparable ischemic event rates and PCI success to DES-only PCI, with a slightly higher, but not statistically significant, rate of major bleeding. Pre-PCI bleeding risk was a strong predictor of post-PCI bleeding, underscoring the importance of individualized risk assessment.

These findings support the potential of DEB-only PCI as a safe, effective, and potentially preferable strategy in high-risk elderly patients, particularly when shorter DAPT is warranted. Further prospective research is needed to confirm these findings and to assess long-term outcomes and cost-effectiveness in this vulnerable population.
